# Geographic Variation in Primary Care Spending Among the Commercially Insured Population

**DOI:** 10.1001/jamanetworkopen.2026.0623

**Published:** 2026-03-05

**Authors:** Kun Li, M. Kate Bundorf, Sara Debab, Rachel Upton, Robert Saunders, Frank McStay

**Affiliations:** 1 Duke-Margolis Institute for Health Policy, Duke University, Washington, DC; 2 Duke-Margolis Institute for Health Policy, Duke University, Durham, North Carolina; 3 Amazon, Seattle, Washington; 4 Sanford School of Public Policy, Duke University, Durham, North Carolina

## Abstract

This cross-sectional study examined the level and variation of primary care spending among the commercially insured population across all core-based statistical area in the US.

## Introduction

Primary care has been the focus of numerous delivery and payment reforms, given its important role in prevention, care coordination, and chronic disease management.^1^ While accurate primary care spending estimates are critical in benchmarking and rate setting, existing data were primarily at the national or state level, offering limited insights into local population profiles and market conditions.^2,3^ We assessed the level and variation of primary care spending among the commercially insured population across all core-based statistical areas (CBSAs) in the US and examined how enrollee demographics and service prices are associated with differences in spending.

## Methods

This cross-sectional study followed the STROBE reporting guideline and was approved by Duke University with a waiver of informed consent. Using the Health Care Cost Institute claims data, we analyzed one-third of US employer-sponsored insurance enrollees aged 64 years or younger with 12-month medical coverage in 2022. Following the literature,^2,4^ we used the narrow definition of primary care spending by measuring enrollees’ annual spending on primary care services rendered by primary care clinicians. Spending included the total amount paid by the insurer and enrollee. Primary care clinicians included those with a code of family practice, internal medicine, pediatric medicine, geriatric medicine, gynecology, physician assistants, or nurse practitioners on more than 50% of professional claims. Primary care services were defined by Current Procedural Terminology (CPT) codes, including evaluation and management visits, preventive visits, care transition or coordination services, and in-office preventive services, screening, and counseling (eMethods in Supplement 1).^5^

We compared 3 CBSA-level mean spending measures—unadjusted, demographic-adjusted (age group and sex), and demographic and price-adjusted (eMethods in Supplement 1). Examining spending at the CBSA level allowed us to capture potential commuting patterns of enrollees for seeking care in urban areas and adjacent communities. To measure cross-CBSA variation in spending, we reported the SD and IQRs of CBSA-level spending. Data were analyzed from October 1, 2024, to March 15, 2025, using Stata SE version 18 (StataCorp).

## Results

Among 25 905 428 enrollees (12 979 555 female [50.1%]) (Table) in 926 CBSAs, mean (SD) annual primary care spending was $282.00 ($383.60) nationally and varied by CBSA, from $112.80 in Vineland-Bridgeton, New Jersey, to $530.30 in Mankato-North Mankato, Minnesota, for unadjusted spending (Figure A). After adjusting for enrollee demographics and service prices, differences in adjusted and unadjusted spending were greater than 10% in 73.0% of CBSAs (Figure B).

**Table.** Characteristics of Enrollees^a^

Characteristic	No. (%)^b^
Total, No.	25 905 428
Sex	
Female	12 979 555 (50.1)
Male	12 925 873 (49.9)
Age group, y	
0-17	5 699 083 (22.0)
18-24	3 010 940 (11.6)
25-34	3 873 647 (15.0)
35-44	4 474 354 (17.3)
45-54	4 523 976 (17.5)
55-64	4 323 428 (17.0)

^a^ Enrollees had 12-month medical coverage and were aged 64 years or younger.

^b^ Percentages may not sum to 100 due to rounding.

**Figure.**
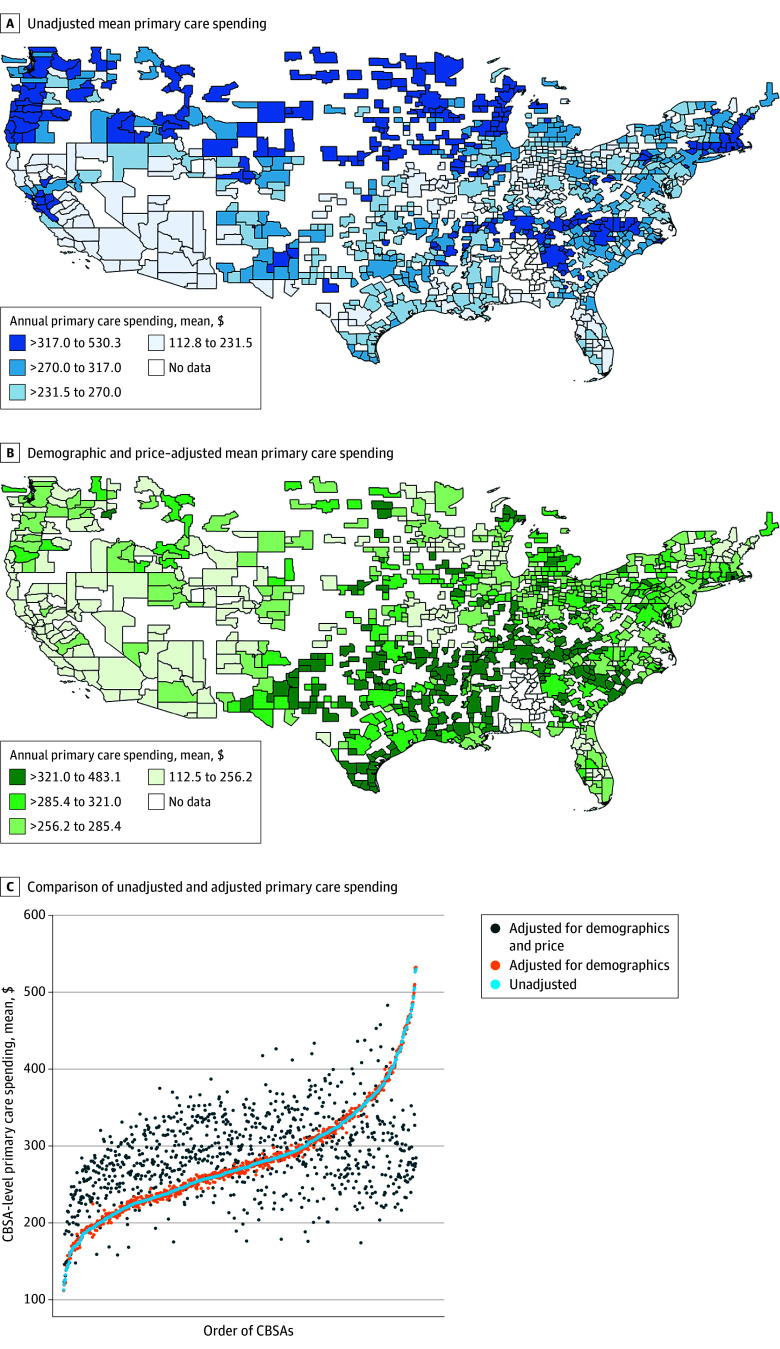
Mean Primary Care Spending by Core-Based Statistical Areas (CBSAs) and Adjusted and Unadjusted Comparisons Mean primary care spending by core-based statistical areas that was (panel A) unadjusted and (panel B) adjusted for enrollee age group, sex, and price. Data for Alabama were suppressed per data contributors. Panel C shows the comparison of mean primary care spending by unadjusted and adjusted CBSAs. Each dot represents a CBSA. The x-axis represents the order of CBSAs by the magnitude of unadjusted mean primary care spending. Data for Alabama and Hawaii were suppressed per data contributors.

Figure C shows unadjusted and adjusted CBSA-level primary care spending, ordered by the magnitude of the unadjusted spending. Demographic-adjusted spending did not largely deviate from unadjusted spending. Cross-CBSA variations were similar for unadjusted spending (median, $270.49; SD, $70.76; IQR, $232.50-$317.00) and demographic-adjusted spending (median, $268.93; SD, $70.80; IQR, $232.50-$316.20). Demographic and price-adjusted spending, in contrast, differed in magnitude and the ordering of CBSAs based on spending levels relative to the unadjusted spending, resulting in lower cross-CBSA variations primarily driven by variations in use (median, $286.84; SD, $54.50; IQR, $257.00-$324.70).

## Discussion

The level of primary care spending varied substantially across CBSAs, regardless of whether adjustments were made for enrollee demographics and service prices. This is consistent with existing evidence on variations in health care spending.^6^ The observed geographic variation in primary care spending was largely due to differences in negotiated price levels rather than variation in enrollees’ age group and sex. Variations in primary care use also contributed to differences in spending.

This study has limitations. Our definition of primary care services may have excluded some primary care clinicians or services. Additionally, the data are not nationally representative.

Our findings are relevant to the design of primary care-focused policies and payment models. By distinguishing the contributions of demographic composition, price, and use, policymakers and payers can identify whether spending differences stem from underlying population needs or market dynamics when setting capitation rates or performance benchmarks for primary care services.

## Notes

Supplement 1.
eMethods.

eReferences.

Supplement 2.
Data Sharing Statement
