# Supplementary material for: Geographic Variation in Primary Care Spending Among the Commercially Insured Population
Source: JAMA Netw Open. 2026 Mar 5;9(3):e260623. doi: 10.1001/jamanetworkopen.2026.0623 (PMC12964154; doi:10.1001/jamanetworkopen.2026.0623)
Supplement: Supplement 1. — eMethods. eReferences. [file jamanetwopen-e260623-s001.pdf]

Supplemental Online Content

Li K, Bundorf MK, Debab S, Upton R, Saunders R, McStay F. Geographic variation in primary care spending among commercially insured population. *JAMA Netw Open*. 2026;9(3):e260623. doi:10.1001/jamanetworkopen.2026.0623

eMethods.

eReferences.

This supplemental material has been provided by the authors to give readers additional information about their work.

© 2026 Li K et al. *JAMA Network Open*.

eMethods

Sample characteristics

We included all enrollees aged < 65 years who had 12-month medical benefit coverage in 2022.

Primary care spending

Our primary care spending measure included spending on primary care services rendered by primary care clinicians in a calendar year. Following a prior study that used the same data source,[1] primary care clinicians were identified by using individual (non-organization) National Provider Identifiers with a provider type code of family practice, internal medicine, pediatric medicine, geriatric medicine, gynecology, physician assistants, or nurse practitioners on more than 50% of professional claims in a year. The provider type code was imputed by the Health Care Cost Institute at the claim level based on the type of services associated with each claim.

Primary care services were identified using the following Current Procedural Terminology codes: 9920x, 9921x, 9924x, 99339-99340, 99341-99345, 99347-99350, 99381-99387, 99391-99397, 99401-99404, 99411-99412, 99420-99429, 99495, 99496, G0402, G0438, G0439.[2] These codes included evaluation and management visits, preventive visits, care transition or coordination services, and in-office preventive services, screening, and counseling.

Demographic-adjusted primary care spending

To calculate demographic-adjusted spending, we first estimated the following linear regression model:

$$Y_i = \alpha \cdot Female_i + \delta \cdot Age_i + \varepsilon_i, \qquad (1)$$

where Y_i is the unadjusted primary care spending for enrollee *i;* $Female_i$ is a binary indicator for sex; Age_i is a set of binary indicators for age groups; ε_i is the residual. For each individual *i*, we estimated predicted spending y_i and residuals $e_i = Y_i - y_i$. We then calculated the demographic-adjusted primary care spending as

$$y_i' = \overline{y_i} + e_i,$$

© 2026 Li K et al. *JAMA Network Open*.

where $\overline{y_i}$ is the national average of predicted spending, which captures the mean spending of a standardized population based on the sex and age group composition of the entire analytic sample; the prediction residual e_i captures variations that cannot be explained by enrollees' sex and age group compositions.

Demographic- and price-adjusted primary care spending

To calculate demographic- and price-adjusted spending, we first calculated price-adjusted primary care spending for each enrollee by using the national average spending for each CPT code. Then, we estimated the linear regression model (1) but used the price-adjusted spending as the outcome. We then computed mean of demographic- and price-adjusted spending following the approach described in the above section.

eReferences

1. Reiff J, Brennan N, Fuglesten Biniek J. Primary Care Spending in the Commercially Insured Population. *JAMA*. 2019;322(22):2244-2245. doi:10.1001/jama.2019.16058

2. Bailit MH, Friedberg MW, Houy ML. Standardizing the Measurement of Commercial Health Plan Primary Care Spending. Milbank Memorial Fund; 2017. Accessed September 1, 2024. https://www.milbank.org/publications/standardizing-measurement-commercial-health-plan-primary-care-spending/

© 2026 Li K et al. *JAMA Network Open*.
