# Supplementary material for: Geographic Variation in Primary Care Spending Among the Commercially Insured Population
Source: JAMA Netw Open. 2026 Mar 5;9(3):e260623. doi: 10.1001/jamanetworkopen.2026.0623 (PMC12964154; doi:10.1001/jamanetworkopen.2026.0623)
Supplement: Supplement 2. — Data Sharing Statement [file jamanetwopen-e260623-s002.pdf]

**Data Sharing Statement**

**Data**
**Data available:** No

**Additional Information**
**Explanation for why data not available:** The data are not publicly available but can be obtained through a data use agreement with the Health Care Cost Institute.
